# Semen abnormalities and associated factors among men seeking fertility services in Eastern and Central Uganda: a multicentre cross-sectional study

**DOI:** 10.1186/s12610-026-00322-4

**Published:** 2026-07-24

**Authors:** Ali Abdow Hafsa, Marie Pascaline Sabine Ishimwe, Musa Kasujja, Maxwell Okello, Theodore Nteziyaremye, Chinyere N. Anyanwu, Sharifah Namutebi, Sara Liban, Ronard Musinguzi, Dale Mugisha, Amina Aden Ahmed, Amina Mohamed Mire, Bahja Ahmed Mumim, Amos Muhumuza, Akib Surat Olabisi, Theoneste Hakizimana

**Affiliations:** 1 Department of Obstetrics and Gynecology, Kampala International University, Bushenyi, Ishaka, Uganda; 2 Department of Pediatrics and Child Health, Kampala International University, Ishaka, Uganda; 3 Department of Sciences, University of Rwanda, Kigali, Rwanda; 4 Department of Obstetrics and Gynecology, Healingway Fertility and Diagnostic Center, Kampala, Uganda; 5 Department of Microbiology, Kampala International University, Ishaka, Uganda; 6 Department of Pathology, Kampala International University, Ishaka, Uganda

**Keywords:** Infertilité masculine, Facteurs de risque, Paramètres du sperme, Antécédents infectieux, Hypertension, Risques professionnels

## Abstract

**Background:**

Male-factor infertility contributes substantially to the burden of infertility, yet evidence on semen abnormalities and their associated factors in Uganda remains limited. This study assessed the prevalence, patterns, and factors associated with abnormal semen values among men seeking fertility services in Eastern and Central Uganda.

**Methods:**

A cross-sectional study was conducted among 155 men attending Jinja Regional Referral Hospital, Kayunga Regional Referral Hospital, and Healingway Diagnostic and Fertility Centre between September 2025 and January 2026. Semen analysis was performed using World Health Organization criteria. Factors associated with abnormal semen values were assessed using bivariable and multivariable logistic regression analyses.

**Results:**

Overall, 52 of 155 participants had at least one abnormal semen parameter, giving a prevalence of 33.5% (95% CI: 26.5–41.4). The prevalence did not differ significantly across study sites (*p* = 0.841). Teratozoospermia (42.3%) and asthenozoospermia (38.5%) were the most common abnormalities, followed by hypospermia (26.9%), necrozoospermia (25.0%), oligozoospermia (23.1%), and azoospermia (23.1%). Hypertension (AOR = 5.23; 95% CI: 1.10–24.93), diabetes mellitus (AOR = 4.82; 95% CI: 1.85–12.57), medication use (AOR = 2.73; 95% CI: 1.11–6.71), history of mumps infection (AOR = 5.29; 95% CI: 1.25–22.31), and occupational hazard exposure (AOR = 4.23; 95% CI: 1.60–11.13) were independently associated with abnormal semen parameters.

**Conclusions:**

Approximately one in three men seeking fertility services in Eastern and Central Uganda had at least one abnormal semen parameter, with abnormalities in sperm morphology and motility being the most common findings. Hypertension, diabetes mellitus, medication use, history of mumps infection, and occupational hazard exposure were associated with abnormal semen parameters. Assessment of medical conditions, previous infections, medication use, and occupational exposures may therefore be useful when evaluating men presenting with infertility.

## Résumé

**Contexte:**

L’infertilité masculine contribue de manière substantielle au fardeau de l’infertilité ; pourtant les preuves concernant les anomalies du sperme et leurs facteurs associés en Ouganda restent limitées. Cette étude a évalué la prévalence, les profils et les facteurs associés à des valeurs anormales de sperme chez les hommes à la recherche de services de fertilité dans l’Est et le Centre de l’Ouganda. Une étude transversale a été réalisée auprès de 155 hommes se présentant à l’hôpital de référence régional Jinja, à l’hôpital régional de référence de Kayunga et au centre de diagnostic et de fertilité de Healingway entre septembre 2025 et janvier 2026. L’analyse du sperme a été réalisée selon les critères de l’Organisation mondiale de la santé. Les facteurs associés à des valeurs anormales des paramètres spermatiques ont été évalués à l’aide d’analyses de régressions logistiques bivariables et multivariables.

**Résultats:**

Au total, 52 participants sur 155 présentaient au moins un paramètre du sperme anormal, soit une prévalence de 33,5 % (IC à 95 % : 26,5–41,4). La prévalence n’a pas différé significativement selon les sites d’étude (p = 0,841). La tératozoospermie (42,3 %) et l’asthénozoospermie (38,5 %) étaient les anomalies les plus courantes, suivies par l’hypospermie (26,9 %), la nécrozoospermie (25,0 %), l’oligozoospermie (23,1 %) et l’azoospermie (23,1 %). L’hypertension (AOR = 5,23 ; IC 95 % : 1,10–24,93), le diabète sucré (AOR = 4,82 ; IC 95 % : 1,85–12,57), l’utilisation de médicaments (AOR = 2,73 ; IC 95 % : 1,11–6,71), les antécédents d’orchites ourliennes (AOR = 5,29 ; IC 95 % : 1,25–22,31) et l’exposition aux risques professionnels (AOR = 4,23 ; IC 95 % : 1,60–11,13) étaient indépendamment associés à des paramètres anormaux du sperme.

**Conclusions:**

Environ un homme sur trois consultant des services de fertilité dans l’Est et le Centre de l’Ouganda présentait au moins un paramètre du sperme anormal, les anomalies de morphologie et de motilité des spermatozoïdes étant les résultats les plus courants. L’hypertension, le diabète sucré, la consommation de médicaments, les antécédents d’orchites ourliennes et l’exposition aux risques professionnels étaient associés à des paramètres anormaux du sperme. L’évaluation des conditions médicales, des infections antérieures, de l’utilisation de médicaments et des expositions professionnelles peuvent donc être utiles lors de l’évaluation des hommes présentant une infertilité.

## Introduction

Infertility is defined as the failure to achieve pregnancy after 12 months or more of regular unprotected sexual intercourse [1]. It affects millions of couples globally and remains an important reproductive health problem with medical, psychological, social, and economic consequences [1, 2]. Male factors contribute to approximately 40%–50% of infertility cases, either alone or together with female factors [2, 3]. Despite this contribution, male infertility is often under-recognized in low-resource settings, where infertility is commonly attributed to women [4, 5]. Semen analysis is the cornerstone of male infertility evaluation because semen quality is a practical indicator of male fecundity [6, 7]. Abnormal semen values such as oligozoospermia, asthenozoospermia, teratozoospermia, azoospermia, and hypospermia reflect impaired male reproductive function and may result from medical, infectious, lifestyle, or environmental causes [7, 8]. According to WHO reference standards, hypospermia is defined as ejaculate volume < 1.5 mL, oligozoospermia as sperm concentration < 15 million/mL, asthenozoospermia as progressive motility < 32%, and teratozoospermia as < 4% normal forms [9, 10].

The prevalence of abnormal semen values varies widely across settings. Reported prevalence include 30.5% to 72.1% in India, 63.0% in Bangladesh, 33.0% in Indonesia, 45.2% in Italy, 38.2% in Nigeria, 84.0% in Ethiopia, 86.8% in Sudan, and 77.0% in Kenya [2, 11–17]. The pattern of abnormalities also differs across studies, although asthenozoospermia, oligozoospermia, teratozoospermia, and azoospermia are most frequently reported [2, 14, 18, 19].

Several factors have been associated with abnormal semen parameters. Lifestyle-related factors such as smoking, alcohol use, and obesity have been linked to reduced semen quality [15, 20–22]. Medical conditions including hypertension and diabetes mellitus have also been associated with semen abnormalities [8, 23]. In addition, occupational heat exposure, chemical exposure, prolonged sitting, pelvic trauma, and previous groin or pelvic surgery have been reported as important contributors [24–27].

Existing evidence suggests that male infertility is often poorly understood, and semen analysis may be underutilized during infertility evaluation [5]. Although abnormal semen parameters are recognized as an important contributor to male infertility, published evidence from Uganda remains limited. In particular, there is limited information on the prevalence, patterns, and factors associated with abnormal semen parameters among men seeking fertility services. Addressing this gap is important because delayed evaluation of male factors may prolong couple distress and delay appropriate management [4, 28]. This study therefore aimed to determine the prevalence, patterns, and factors associated with abnormal semen parameters among men seeking fertility services at Jinja Regional Referral Hospital, Kayunga Regional Referral Hospital, and Healingway Diagnostic and Fertility Centre.

## Participants and methods

### Study design and settings

This hospital-based cross-sectional analytical study was conducted among men seeking fertility services at Jinja Regional Referral Hospital (JRRH), Kayunga Regional Referral Hospital (KRRH), and Healingway Diagnostic and Fertility Centre in Eastern and Central Uganda. The study was carried out from 17th September 2025 to 17th January 2026. The design enabled estimation of the prevalence of abnormal semen quality, description of the common semen abnormalities, and assessment of factors associated with abnormal semen values among men presenting for infertility evaluation. JRRH is a major regional referral hospital located in Jinja City and serves patients from Jinja and surrounding districts. KRRH serves as a referral facility for Kayunga District and nearby communities, while Healingway Diagnostic and Fertility Centre provides specialized fertility diagnostic and management services. Together, these facilities provided suitable sites for studying semen abnormalities among men seeking fertility care in Eastern and Central Uganda.

### Study population

The study population comprised men seeking fertility services at Jinja Regional Referral Hospital, Kayunga Regional Referral Hospital, and Healingway Diagnostic and Fertility Centre during the study period. Men were eligible if they were attending the selected facilities for fertility evaluation and provided written informed consent. Men who were unable to provide a semen sample or had conditions that made participation impossible were excluded.

### Sample size calculation

The sample size was calculated using the Kish–Leslie formula for estimating a single population proportion:

$$\:no=\frac{{Z}^{2}p(1-p)}{{d}^{2}}$$

Where n₀ is the initial sample size, Z is the standard normal value corresponding to a 95% confidence level (1.96), p is the estimated prevalence of the most common abnormal semen parameter from a previous study in Sudan (26.41%) [18] and d is the margin of error set at 5%.

Using these assumptions:

$$\mathrm{n}_{0}=\left(1.96^{2}\times0.2641\times\left[1{-}0.2641\right]\right)/0.05^2=299$$

Because the estimated number of men expected to attend fertility services across the three study sites during the study period was approximately 200, a finite population correction was applied:

$$\mathrm{n}=\mathrm{n}_{0}/\left[1+\left\{\left(\mathrm{n}_{0}-1\right)/\mathrm{N}\right\}\right]$$

$$\mathrm{n}=299/\left[1+\left\{\left(299-1\right)/200\right\}\right]=120$$

After adding 10% to account for possible non-response or incomplete semen samples, the minimum required sample size was 132 participants. However, 155 eligible men consented and were included in the final analysis.

### Sampling technique

Eligible men seeking fertility services at the three study sites were recruited consecutively until the required sample size was achieved. Each man was assessed for eligibility, informed about the study, and enrolled after providing written informed consent. Recruitment continued at all participating sites until the final sample size was attained. After enrollment, each participant completed a structured interviewer-administered questionnaire and provided a semen sample for laboratory analysis.

### Data collection procedure

Eligible men attending fertility services at the selected study sites were approached by the research team, provided with information about the study, and enrolled after giving written informed consent in either English or the local language. Data were collected using a structured interviewer-administered questionnaire that was developed from previous studies and adapted to the study setting. The questionnaire collected information on sociodemographic characteristics, lifestyle factors, medical history, sexual and reproductive history, and occupational or environmental exposures. Before the start of data collection, the questionnaire was pretested among men attending a similar health facility that was not included in the study, and minor revisions were made to improve clarity and ensure consistency. Trained research assistants administered the questionnaire using standardized procedures.

After the interview, participants were guided on semen sample collection procedures and advised to observe the recommended period of sexual abstinence before providing a specimen. Semen samples were collected by masturbation into sterile containers in a private room within the study facility and transported promptly to the laboratory for analysis. Laboratory assessment was performed according to World Health Organization (WHO) recommendations. Participants were informed of their results, and those with abnormal findings were advised to seek further clinical evaluation and appropriate follow-up care.

### Study variables

The independent variables in this study included socio-demographic, behavioral, medical, sexual, and environmental factors considered to be associated with abnormal semen values among men seeking fertility services. These included age, marital status, education level, occupation, residence, smoking, alcohol use, body mass index, hypertension, diabetes mellitus, and history of mumps infection, medication use, and duration of infertility, sexual abstinence period, previous pelvic or groin surgery, trauma, and occupational hazard exposure.

The dependent variable was abnormal semen parameters, determined by semen analysis using standard WHO criteria. A participant was considered to have abnormal semen values if at least one semen parameter was outside the WHO [10] reference range.

Medication use referred to self-reported use of prescribed medications for chronic or acute medical conditions within the preceding three months.

Occupational hazard exposure included self-reported exposure to heat, pesticides, industrial chemicals, fuels, radiation, dust, prolonged sitting, or other workplace conditions considered potentially harmful to reproductive health.

For epidemiological purposes, abnormal semen values were defined as the presence of at least one semen parameter outside the WHO reference range. This composite outcome captures heterogeneous abnormalities and was used to estimate the overall burden of impaired semen quality. Specific semen abnormalities were therefore described descriptively rather than modeled separately, because several abnormalities co-occurred and the number of participants within individual abnormality subgroups was limited.

### Semen analysis

Participants were instructed to abstain from ejaculation for 2–7 days before semen collection in accordance with WHO [10] recommendations. Semen samples were obtained by masturbation into sterile, wide-mouthed containers in a private room within the study facility. The time of sample collection was recorded, and the specimens were delivered immediately to the laboratory for analysis.

Upon receipt, samples were allowed to liquefy at room temperature before examination. Semen analysis was performed according to the WHO Laboratory Manual for the Examination and Processing of Human Semen [10]. The parameters assessed included semen volume, sperm concentration, total sperm count, motility, morphology, vitality. Semen parameter values falling outside the WHO reference limits were classified as abnormal. Seminal pH values below 7.2 or above 8.0 were considered abnormal according to WHO reference limits. All analyses were performed by trained laboratory personnel using standardized procedures.

### Quality control

Several measures were taken to support the quality of the data collected during the study. Before data collection began, the study tools and procedures were pretested to assess clarity, consistency, and suitability for the study setting. Research assistants received training on the study objectives, participant recruitment, informed consent procedures, and administration of the questionnaire. Throughout the study period, completed questionnaires were reviewed for completeness and consistency, and any identified issues were addressed promptly.

Semen samples were labeled using unique study identification numbers to ensure correct specimen identification and maintain participant confidentiality. Routine checks were conducted to verify specimen labeling, sample handling, and adherence to laboratory procedures. Laboratory activities were supervised periodically throughout the study to ensure consistency in sample processing and reporting of results.

### Data management and analysis

Data were collected using coded interviewer-administered questionnaires and laboratory result forms, then securely stored in locked file boxes. Electronic data were entered into Microsoft Excel, cleaned, backed up regularly, and password-protected before being exported to STATA version 14.2 for statistical analysis.

Categorical variables were summarized using frequencies and percentages, whereas continuous variables were summarized using means and standard deviations (SDs) for normally distributed data or medians and interquartile ranges (IQRs) for skewed data. Statistical significance was set at *p* < 0.05, and 95% confidence intervals (CIs) were reported where applicable.

The prevalence of abnormal semen values was calculated as the proportion of men with at least one abnormal semen parameter out of all study participants and presented as a percentage with corresponding 95% confidence intervals. The patterns of semen abnormalities were determined from laboratory findings among participants with abnormal semen results. The proportions of teratozoospermia, asthenozoospermia, hypospermia, necrozoospermia, oligozoospermia, azoospermia, and other abnormalities were then calculated among men with abnormal semen parameters.

To assess factors associated with abnormal semen parameters, bivariate logistic regression analysis was first performed for each independent variable. Crude odds ratios (cORs), 95% confidence intervals, and p-values were reported. Variables with *p* < 0.20 at bivariate analysis, together with those considered biologically plausible, were included in the multivariable logistic regression model to identify factors independently associated with abnormal semen parameters. Adjusted odds ratios (aORs), 95% confidence intervals, and p-values were reported. A p-value < 0.05 was considered statistically significant in the final adjusted analysis.

### Human ethics and consent to participate

This study was approved by the Research Ethics Committee of Kampala International University and the administrations of Jinja Regional Referral Hospital, Kayunga Regional Referral Hospital, and Healingway Diagnostic and Fertility Centre under registration number KIU-REC-2025-1733. The study was subsequently registered with the Uganda National Council for Science and Technology (UNCST). All participants provided written informed consent before enrollment after receiving information about the purpose, procedures, potential benefits, and risks of the study. Consent was obtained in either English or the local language to ensure adequate understanding and voluntary participation. Participants were informed of their right to decline participation or withdraw from the study at any stage without affecting the care they received. Confidentiality was maintained throughout the study through the use of unique study identification numbers in place of participant names. All study records and laboratory results were stored securely and were accessible only to authorized members of the research team. The study was conducted in accordance with the ethical principles outlined in the Declaration of Helsinki.

## Results

### Basic characteristics of participants

**Fig. 1:**
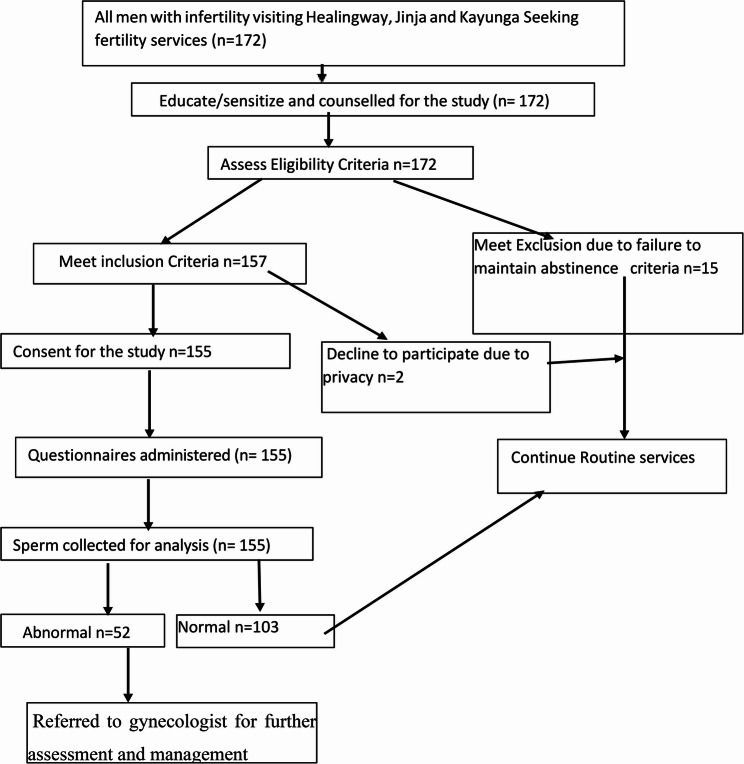
Study flow diagram of participant recruitment and enrolment across the three study sites (*N* = 155)

Among the 155 men enrolled in this study (Fig. 1), the majority were aged 31–40 years, 62 (40.0%), had attained tertiary education, 76 (49.0%), and were formally employed, 91 (58.7%). Most participants had no history of smoking, 109 (70.3%), and no history of alcohol intake, 101 (65.2%), while 85 (54.8%) were physically active. With respect to clinical characteristics, most participants had normal body mass index, 71 (45.8%), whereas 50 (32.3%) were overweight, 25 (16.1%) were obese, and 9 (5.8%) were underweight. The majority had no history of hypertension, 140 (90.3%), no history of diabetes mellitus, 123 (79.4%), and no history of sleeping disorders, 128 (82.6%). Most participants were not using any medication, 124 (80.0%), and 140 (90.3%) had no history of mumps infection. More than half of the participants had never fathered a child, 86 (55.5%), while 61 (39.4%) reported occupational hazard exposure. Most participants had no previous history of surgical procedure or pelvic trauma, 136 (87.7%) (Table 1).

**Table 1:** Sociodemographic, medical, and reproductive characteristics of study participants by study site (*N* = 155)

Characteristic	Overall *N* = 155 (%)	Jinja *n* = 26 *n* (%)	Kayunga *n* = 24 *n* (%)	Healingway *n* = 105 *n* (%)	χ²(df), *p*
Age (years)					Fisher’s exact *p* = 0.615
20–30	43 (27.7)	8 (30.8)	4 (16.7)	31 (29.5)	
31–40	62 (40.0)	12 (46.2)	10 (41.7)	40 (38.1)	
41–50	34 (21.9)	3 (11.5)	8 (33.3)	23 (21.9)	
≥ 51	16 (10.3)	3 (11.5)	2 (8.3)	11 (10.5)	
Educational level					Fisher’s exact *p* < 0.001
No formal	6 (3.9)	3 (11.5)	3 (12.5)	0 (0.0)	
Primary	14 (9.0)	5 (19.2)	9 (37.5)	0 (0.0)	
Secondary	59 (38.1)	10 (38.5)	6 (25.0)	43 (41.0)	
Tertiary	76 (49.0)	8 (30.8)	6 (25.0)	62 (59.0)	
Occupation					Fisher’s exact *p* < 0.001
Unemployed	13 (8.4)	9 (34.6)	4 (16.7)	0 (0.0)	
Informal	51 (32.9)	11 (42.3)	10 (41.7)	30 (28.6)	
Formal	91 (58.7)	6 (23.1)	10 (41.7)	75 (71.4)	
History of smoking					χ²(2) = 0.37, *p* = 0.832
Yes	46 (29.7)	9 (34.6)	7 (29.2)	30 (28.6)	
No	109 (70.3)	17 (65.4)	17 (70.8)	75 (71.4)	
History of alcohol intake					χ²(2) = 1.22, *p* = 0.544
None	101 (65.2)	19 (73.1)	14 (58.3)	68 (64.8)	
Occasional	54 (34.8)	7 (26.9)	10 (41.7)	37 (35.2)	
Physical activity					χ²(2) = 1.99, *p* = 0.369
Active	85 (54.8)	11 (42.3)	14 (58.3)	60 (57.1)	
Not active	70 (45.2)	15 (57.7)	10 (41.7)	45 (42.9)	
Body Mass Index (WHO)					Fisher’s exact *p* = 0.157
Underweight	9 (5.8)	0 (0.0)	0 (0.0)	9 (8.6)	
Normal	71 (45.8)	8 (30.8)	14 (58.3)	49 (46.7)	
Overweight	50 (32.3)	12(46.2)	7 (29.2)	31 (29.5)	
Obese	25 (16.1)	6 (23.1)	3 (12.5)	16 (15.2)	
Hypertension					Fisher’s exact *p* = 0.009
Yes	15 (9.7)	0 (0.0)	6 (25.0)	9 (8.6)	
No	140(90.3)	26(100.0)	18 (75.0)	96 (91.4)	
History of diabetes					χ²(2) = 0.04, *p* = 0.981
Yes	32 (20.6)	5(19.2)	5 (20.8)	22 (21.0)	
No	123(79.4)	21(80.8)	19 (79.2)	83 (79.0)	
History of sleeping disorders					χ²(2) = 3.08, *p* = 0.215
Yes	27 (17.4)	5 (19.2)	7 (29.2)	15 (14.3)	
No	128(82.6)	21(80.8)	17 (70.8)	90 (85.7)	
Medication use					Fisher’s exact *p* = 0.589
Yes	31 (20.0)	6 (23.1)	3 (12.5)	22 (21.0)	
No	124(80.0)	20 (76.9)	21 (87.5)	83 (79.0)	
Mumps history					Fisher’s exact *p* = 0.844
Yes	15 (9.7)	2 (7.7)	3 (12.5)	10 (9.5)	
No	140(90.3)	24 (92.3)	21 (87.5)	95 (90.5)	
Ever fathered					χ²(2) = 0.44, *p* = 0.804
Yes	69 (44.5)	12 (46.2)	12 (50.0)	45 (42.9)	
No	86 (55.5)	14 (53.8)	12 (50.0)	60 (57.1)	
Occupational hazards					χ²(2) = 4.85, *p* = 0.089
Yes	61 (39.4)	15 (57.7)	10 (41.7)	36 (34.3)	
No	94 (60.6)	11 (42.3)	14 (58.3)	69 (65.7)	
Previous history of surgical procedure / pelvic trauma					Fisher’s exact *p* = 0.791
Yes	19 (12.3)	3 (11.5)	2 (8.3)	14 (13.3)	
No	136 (87.7)	23 (88.5)	22 (91.7)	91 (86.7)	

### Prevalence and patterns of abnormal semen values among men seeking fertility services in Eastern and Central Uganda

**Fig. 2:**
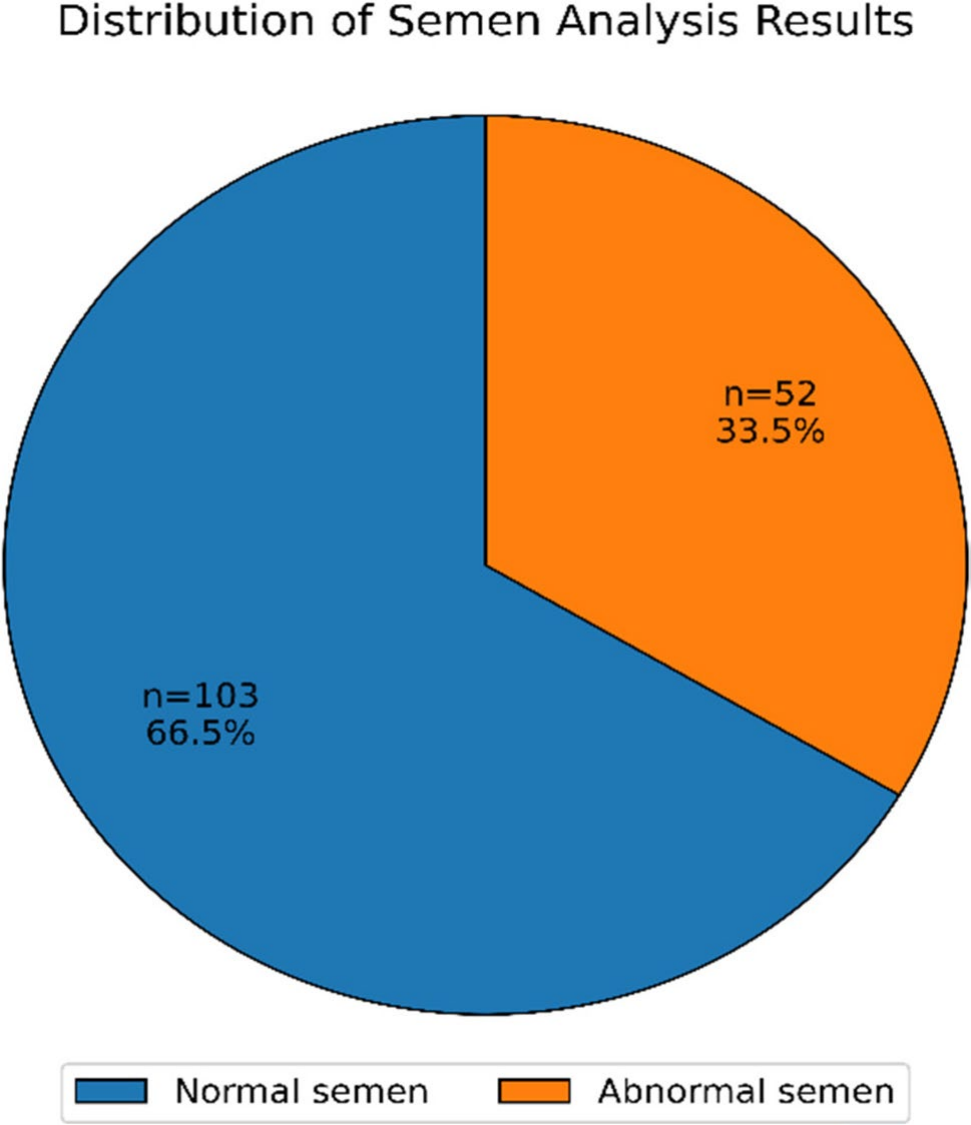
Prevalence of abnormal semen values among men seeking fertility services in Eastern and Central Uganda (*N* = 155). Percentages were calculated using the total study population as the denominator.

The prevalence of abnormal semen values among study participants was 33.5% (95% CI: 26.5%–41.4%) (Fig. 2). Among participants with abnormal semen findings, teratozoospermia and asthenozoospermia were the most common abnormalities (Table 2).

**Table 2:** Distribution of abnormal semen values among participants with abnormal semen findings across the three study sites (*n* = 52). Percentages within each study site were calculated using the number of participants with abnormal semen values at that site as the denominator

Abnormal semen parameter	Overall *n* (%)	Jinja *n* = 10 n (%)	Kayunga *n* = 8 n (%)	Healingway *n* = 34 n (%)
Hypospermia	14 (26.9)	3 (30.0)	2 (25.0)	9 (26.5)
Azoospermia	12 (23.1)	3 (30.0)	2 (25.0)	7 (20.6)
Oligozoospermia	12 (23.1)	4 (40.0)	2 (25.0)	6 (17.6)
Asthenozoospermia	20 (38.5)	8 (80.0)	3 (37.5)	9 (26.5)
Teratozoospermia	22 (42.3)	6 (60.0)	5 (62.5)	11 (32.4)
Necrozoospermia	13 (25.0)	4 (40.0)	3 (37.5)	6 (17.6)

### Factors associated with abnormal semen values among men seeking fertility services in Eastern and Central Uganda

At bivariate analysis, age group, smoking status, physical activity, body mass index, hypertension, diabetes mellitus, history of sleeping disorders, current medication use, sexual intercourse frequency, ever fathered a pregnancy, and occupational hazard exposure had p-values < 0.20 and therefore met the pre-specified criterion for inclusion in the multivariable logistic regression model (Table 3). After adjusting for potential confounders in the multivariable logistic regression model, five factors remained statistically significant independent factors of abnormal semen parameters. History of mumps infection was the strongest factor (aOR = 5.29, 95% CI: 1.25–22.31), followed by hypertension (aOR = 5.23, 95% CI: 1.10–24.93), diabetes mellitus (aOR = 4.82, 95% CI: 1.85–12.57), occupational hazard exposure (aOR = 4.23, 95% CI: 1.60–11.13), and current medication use (aOR = 2.73, 95% CI: 1.11–6.71) (Table 4).

**Table 3:** Bivariable logistic regression analysis of factors associated with abnormal semen values among men seeking fertility services in Eastern and Central Uganda (*N* = 155)

Characteristic	Normal semen (*n* = 103), n (%)	Abnormal semen (*n* = 52), n (%)	COR	95% CI	*p*-value
Age group (years)					
20–30 (Ref)	34 (33.0)	9 (17.3)	Ref		
31–40	45 (43.7)	17 (32.7)	1.43	0.57–3.60	0.451
41–50	18 (17.5)	16 (30.8)	3.36	1.24–9.13	**0.018**
≥ 51	6 (5.8)	10 (19.2)	6.30	1.80-22.08	**0.004**
Highest education level					
No formal education	3 (2.9)	3 (5.8)	1.92	0.36–10.26	0.444
Primary	8 (7.8)	6 (11.5)	1.44	0.45–4.62	0.537
Secondary	42 (40.8)	17 (32.7)	0.78	0.37–1.63	0.506
Tertiary (Ref)	50 (48.5)	26 (50.0)	Ref		
Occupation type					
Formal (Ref)	57 (55.3)	34 (65.4)	Ref		
Informal	36 (35.0)	15 (28.8)	0.70	0.33–1.46	0.342
Unemployed	10 (9.7)	3 (5.8)	0.50	0.13–1.96	0.323
Smoking status					
No (Ref)	78 (75.7)	31 (59.6)	Ref		
Yes	25 (24.3)	21 (40.4)	2.11	1.03–4.33	**0.041**
Alcohol consumption					
None (Ref)	65 (63.1)	36 (69.2)	Ref		
Occasional	38 (36.9)	16 (30.8)	0.76	0.37–1.55	0.452
Physical activity					
Yes (Ref)	50 (48.5)	35 (67.3)	Ref		
No	53 (51.5)	17 (32.7)	0.46	0.23–0.92	**0.029**
Body mass index (WHO)					
Normal (Ref)	53 (51.5)	18 (34.6)	Ref		
Underweight	6 (5.8)	3 (5.8)	1.47	0.33–6.53	0.611
Overweight	32 (31.1)	18 (34.6)	1.66	0.75–3.65	0.210
Obese	12 (11.7)	13 (25.0)	3.19	1.23–8.27	**0.017**
Hypertension					
No (Ref)	96 (93.2)	44 (84.6)	Ref		
Yes	7 (6.8)	8 (15.4)	2.49	0.85–7.33	**0.097**
Diabetes mellitus					
No (Ref)	90 (87.4)	33 (63.5)	Ref		
Yes	13 (12.6)	19 (36.5)	3.99	1.77–8.99	**0.001**
History of sleeping disorders					
No (Ref)	89 (86.4)	39 (75.0)	Ref		
Yes	14 (13.6)	13 (25.0)	2.12	0.91–4.94	0.082
Current medication use					
No (Ref)	86 (83.5)	38 (73.1)	Ref		
Yes	17 (16.5)	14 (26.9)	1.86	0.83–4.17	**0.130**
History of mumps infection					
No (Ref)	95 (92.2)	45 (86.5)	Ref		
Yes	8 (7.8)	7 (13.5)	1.85	0.63–5.43	0.265
Sexual intercourse frequency					
≥ 4/week (Ref)	53 (51.5)	21 (40.4)	Ref		
< 4/week	50 (48.5)	31 (59.6)	1.56	0.79–3.08	**0.195**
Ever fathered a pregnancy					
Yes (Ref)	51 (49.5)	18 (34.6)	Ref		
No	52 (50.5)	34 (65.4)	1.85	0.93–3.70	**0.081**
Occupational hazard exposure					
No (Ref)	71 (68.9)	23 (44.2)	Ref		
Yes	32 (31.1)	29 (55.8)	2.80	1.40–5.58	**0.003**
Previous pelvic or reproductive surgery					
No (Ref)	90 (87.4)	46 (88.5)	Ref		
Yes	13 (12.6)	6 (11.5)	0.90	0.32–2.54	0.847

Bold are *p* values < 0.2

**Table 4:** Multivariable logistic regression analysis of factors associated with abnormal semen values among men seeking fertility services in Eastern and Central Uganda (*N* = 155)

Characteristic	COR	95% CI	*p*-value	AOR	95% CI	*p*-value
Age group (years)						
20–30 (Ref)	Ref			Ref		
31–40	1.43	0.57–3.60	0.451	0.79	0.24–2.57	0.693
41–50	3.36	1.24–9.13	0.018	2.73	0.73–10.26	0.136
≥ 51	6.30	1.80-22.08	0.004	3.33	0.65–17.10	0.149
Smoking status						
No (Ref)	Ref			Ref		
Yes	2.11	1.03–4.33	0.041	2.09	0.88–4.94	0.093
Physical activity						
Yes (Ref)	Ref			Ref		
No	0.46	0.23–0.92	0.029	0.52	0.20–1.32	0.168
Body mass index (WHO)						
Normal (Ref)	Ref			Ref		
Underweight	1.47	0.33–6.53	0.611	1.19	0.16–8.75	0.862
Overweight	1.66	0.75–3.65	0.210	0.95	0.36–2.49	0.915
Obese	3.19	1.23–8.27	0.017	3.00	0.86–10.45	0.085
Hypertension						
No (Ref)	Ref			Ref		
Yes	2.49	0.85–7.33	0.097	5.23	1.10-24.93	**0.038**
Diabetes mellitus						
No (Ref)	Ref			Ref		
Yes	3.99	1.77–8.99	0.001	4.82	1.85–12.57	**0.001**
History of sleeping disorders						
No (Ref)	Ref			Ref		
Yes	2.12	0.91–4.94	0.082	1.65	0.57–4.77	0.359
Current medication use						
No (Ref)	Ref			Ref		
Yes	1.86	0.83–4.17	0.130	2.73	1.11–6.71	**0.029**
History of mumps infection						
No (Ref)	Ref			Ref		
Yes	1.85	0.63–5.43	0.265	5.29	1.25–22.31	**0.023**
Sexual intercourse frequency						
≥ 4/week (Ref)	Ref			Ref		
< 4/week	1.56	0.79–3.08	0.195	1.16	0.47–2.85	0.747
Ever fathered a pregnancy						
Yes (Ref)	Ref			Ref		
No	1.85	0.93–3.70	0.081	1.40	0.54–3.67	0.490
Occupational hazard exposure						
No (Ref)	Ref			Ref		
Yes	2.80	1.40–5.58	0.003	4.23	1.60-11.13	**0.004**

Crude Odds Ratios (COR) were obtained from bivariate logistic regression, while Adjusted Odds Ratios (AOR) were obtained from multivariable logistic regression using robust standard errors. Statistical significance was set at *p* < 0.05

Bold are *p* values <0.05

## Discussion

This study found that 33.5% of men seeking fertility services had at least one abnormal semen value. The prevalence was similar across Jinja Regional Referral Hospital, Kayunga Regional Referral Hospital, and Healingway Diagnostic and Fertility Centre, suggesting that abnormal semen findings were observed across the participating facilities.

The prevalence observed in this study was lower than that reported in several studies from Kenya, Ethiopia, Nigeria, Cameroon, and Ghana, where prevalence estimates ranged from 69.1% to 84.3%. [14, 17, 29–32]. However, comparisons across studies should be interpreted with caution because of differences in study populations, referral settings, and approaches used to assess semen quality. Many of these studies were conducted in tertiary or specialist infertility centers, which may receive men with longer-standing or more complex fertility problems than those attending routine fertility services [14, 17]. Such differences in case mix may partly explain the higher prevalence estimates reported in those settings.

In contrast, the prevalence observed in the present study was closer to findings from less selective or mixed clinical populations, including reports of 38.2% in Nigeria and 32% abnormal sperm density in another Nigerian study [24, 33]. Similar prevalence estimates have also been reported from Sri Lanka and Indonesia [34, 35]. Although direct comparisons remain challenging, these findings suggest that the burden observed in Eastern and central Uganda may be comparable to that reported in other routine fertility-care settings.

Methodological differences may also have contributed to the variation in prevalence estimates across studies. For example, Ngwu et al. performed two semen analyses per participant and reported a prevalence of 66.5% [36], whereas older Nigerian data reported a prevalence of 27.3% using earlier WHO reference criteria [37]. In addition, studies have differed in the number of semen samples analyzed, laboratory procedures used, and definitions applied to classify abnormal findings. Taken together, these differences highlight the need for caution when comparing prevalence estimates across settings and underscore the importance of considering study design and methodology when interpreting the burden of abnormal semen parameters.

Among men with abnormal semen findings, teratozoospermia and asthenozoospermia were the most common patterns. This indicates that defects in sperm morphology and motility were the dominant abnormalities in this population. Such findings are clinically important because both parameters are sensitive to infection, inflammation, oxidative stress, metabolic disease, and environmental exposures [14, 17].

The prominence of motility-related abnormalities is consistent with findings from Kenya, Ethiopia, Bangladesh, and India, where asthenozoospermia was also commonly reported as a leading abnormality [2, 14, 15, 17, 19]. This suggests that impaired motility may be a common expression of male reproductive dysfunction across different infertility settings.

The high contribution of teratozoospermia in the present study may reflect both true biological impairment and known variability in morphology assessment. Morphology is one of the most observer-dependent semen parameters and may vary across laboratories depending on staining technique, technician experience, and strictness of WHO criteria application [17]. This may explain why the differing relative frequency of teratozoospermia across studies.

Other abnormalities identified in this study included hypospermia, necrozoospermia, oligozoospermia, and azoospermia, showing that semen impairment in this population was not limited to a single defect. Similar multi-pattern distributions have been described in Ethiopian and Nigerian studies, although the exact dominant abnormality varies by setting and referral profile [17, 32, 36].

This study found that hypertension, diabetes mellitus, current medication use, history of mumps infection, and occupational hazard exposure were independently associated with abnormal semen parameters. These findings suggest that semen abnormalities in this population are strongly linked to chronic disease, prior reproductive injury, and environmental exposure. Given the cross-sectional design and the absence of several potentially important reproductive and lifestyle variables, the observed associations should be interpreted as exploratory correlates rather than definitive independent determinants of impaired semen quality.

The associations with hypertension and diabetes mellitus are biologically plausible. Cardiometabolic disease may impair semen quality through oxidative stress, hormonal dysregulation, endothelial dysfunction, and reduced testicular microcirculation [38, 39]. Similar associations have been reported in studies from Africa and elsewhere, supporting the role of chronic non-communicable disease in male infertility [17, 29, 31].

Medication use was also independently associated with abnormal semen parameters. In this setting, medication use is likely a marker of underlying chronic illness rather than an isolated exposure. This interpretation is consistent with the observed importance of hypertension and diabetes in the same model and with previous evidence that treatment variables often reflect background disease burden [36].

A history of mumps infection remained an independent factor of abnormal semen parameters. This is consistent with established evidence that post-pubertal mumps may damage the testes, especially through orchitis, and lead to long-term impairment of sperm production [36]. This finding highlights the continuing reproductive consequences of preventable infections.

Occupational hazard exposure was another important independent factor. This is plausible in a setting where many men may be exposed to heat, dust, chemicals, fuels, pesticides, or prolonged physical strain at work. Evidence from Cameroon and other settings has linked occupational and environmental exposures to poor semen quality, especially abnormalities in motility, morphology, and viability [30, 40]. Occupational hazard exposure in this study was a broad self-reported category that included heat, pesticides, industrial chemicals, fuels, radiation, dust, prolonged sitting, and related workplace conditions. These exposures are biologically distinct and may not affect semen quality in the same way; therefore, the observed association should be interpreted as a general indicator of workplace-related risk rather than the effect of any single exposure.

Some variables, including age, body mass index, and smoking, showed crude associations but did not remain significant after adjustment. This suggests that their effects may be mediated through other factors such as cardiometabolic disease or occupational exposure. Similar attenuation after multivariable adjustment has been reported in other studies [38, 41].

Overall, these findings indicate that abnormal semen values in this population are associated more strongly with clustered medical and environmental risk factors than with isolated demographic characteristics. Clinically, this supports routine assessment of chronic disease, infection history, medication exposure, and occupational hazards during infertility evaluation. In addition, emerging evidence suggests that modifiable lifestyle factors, including dietary quality, may influence semen quality. Healthier dietary patterns, particularly Mediterranean-style diets, have been associated with better semen profiles, whereas poorer dietary patterns may contribute to impaired semen parameters. Future studies in this setting should therefore consider dietary assessment alongside medical and occupational risk factors [42, 43].

### Strengths and limitations

This multicenter study provides useful data on semen abnormalities among men seeking fertility care in Eastern and Central Uganda and used standardized WHO-based semen analysis. However, the cross-sectional design does not allow causal or temporal interpretation. Semen analysis was based on a single specimen, and because semen quality varies over time, the prevalence estimates and observed associations should be interpreted cautiously. In addition, the primary outcome grouped heterogeneous abnormalities, so the reported prevalence reflects the overall burden of impaired semen quality rather than a single clinical entity. Some important factors such as varicocele, hormonal profile, genital infections, diet, stress, genetic abnormalities, and sperm DNA fragmentation were not assessed, leaving the possibility of residual confounding. Occupational exposure was also measured broadly and may represent different biological risks. Finally, some adjusted estimates had wide confidence intervals, and because participants were recruited from fertility clinics, the findings may not be generalizable beyond similar clinical settings.

## Conclusions and recommendations

Approximately one in three men seeking fertility services in Eastern and Central Uganda had at least one abnormal semen parameter, with teratozoospermia and asthenozoospermia being the most common abnormalities. Hypertension, diabetes mellitus, medication use, history of mumps infection, and occupational hazard exposure were independently associated with abnormal semen parameters. These findings support routine semen analysis and careful assessment of medical history and occupational exposures during male infertility evaluation in similar settings.

## Data Availability

All data underlying the findings are fully available without restriction. The de-identified dataset are provided as Supporting Information files.

## Abbreviations

aOR: Adjusted Odds Ratio
HIV: Human Immune Virus
KIU-TH: Kampala International University Teaching Hospital
PI: Principal Investigator
WHO: World Health Organization
Gyn: Gynecology
